# Seasonal Mass Changes and Crustal Vertical Deformations Constrained by GPS and GRACE in Northeastern Tibet

**DOI:** 10.3390/s16081211

**Published:** 2016-08-02

**Authors:** Yuanjin Pan, Wen-Bin Shen, Cheinway Hwang, Chaoming Liao, Tengxu Zhang, Guoqing Zhang

**Affiliations:** 1 School of Geodesy and Geomatics, Wuhan University, Wuhan 430079, China; pan_yuanjin@163.com (Y.P.); cheinway@mail.nctu.edu.tw (C.H.); txzhang521@163.com (T.Z.); gqzhangsgg@whu.edu.cn (G.Z.); 2 State Key Laboratory of Information Engineering in Surveying, Mapping and Remote Sensing, Wuhan University, Wuhan 430079, China; 3 Department of Civil Engineering, National Chiao Tung University, Hsinchu 300, Taiwan; 4 School of Land Resources and Surveying, Guangxi Teachers Education University, Nanning 530001, China; gps94041@163.com

**Keywords:** CGPS time series, GRACE observations and surface loads, empirical orthogonal function, crustal vertical deformation

## Abstract

Surface vertical deformation includes the Earth’s elastic response to mass loading on or near the surface. Continuous Global Positioning System (CGPS) stations record such deformations to estimate seasonal and secular mass changes. We used 41 CGPS stations to construct a time series of coordinate changes, which are decomposed by empirical orthogonal functions (EOFs), in northeastern Tibet. The first common mode shows clear seasonal changes, indicating seasonal surface mass re-distribution around northeastern Tibet. The GPS-derived result is then assessed in terms of the mass changes observed in northeastern Tibet. The GPS-derived common mode vertical change and the stacked Gravity Recovery and Climate Experiment (GRACE) mass change are consistent, suggesting that the seasonal surface mass variation is caused by changes in the hydrological, atmospheric and non-tidal ocean loads. The annual peak-to-peak surface mass changes derived from GPS and GRACE results show seasonal oscillations in mass loads, and the corresponding amplitudes are between 3 and 35 mm/year. There is an apparent gradually increasing gravity between 0.1 and 0.9 μGal/year in northeast Tibet. Crustal vertical deformation is determined after eliminating the surface load effects from GRACE, without considering Glacial Isostatic Adjustment (GIA) contribution. It reveals crustal uplift around northeastern Tibet from the corrected GPS vertical velocity. The unusual uplift of the Longmen Shan fault indicates tectonically sophisticated processes in northeastern Tibet.

## 1. Introduction

Mass changes in and around the Tibet Plateau represent a hot researched and complicated issue in the geophysical and Earth science communities because of the crustal motion, geological structures, and seasonal fluctuations of surface deformation. The ongoing underthrusting of the Indian plate beneath the Eurasian plate not only uplifted the Himalayas, the highest mountains in the world, but also produced hundreds of fault zones in and around the boundaries of the Tibetan Plateau [1,2]. These rapid tectonic movements could be related to the current orogenesis caused by India–Eurasia collision or flow in the asthenosphere related to the absolute motion of Eurasia [3]. Evidence suggests that the Indian crust retains its strength as it underthrusts the plateau [4]. However, how the crustal vertical deformation varies and the mechanism of underlying material changes in and around the Tibetan Plateau remains unclear.

Present-day Global Positioning System (GPS)-derived horizontal velocities indicate that the Asian mainland plate is rotating clockwise and that the Indian plate is pushing the Eurasian plate along the Himalayas in the NNE direction, causing the Eastern Himalayan Syntaxis to rotate clockwise and resulting in a fan-like front at the southeast corner of the plateau [2,5,6,7,8]. Most studies have focused on the horizontal crustal motion in and around Tibet. However, due to the limited resolutions and accuracies in the geophysical corrections of GPS-derived vertical deformation, GPS vertical velocity estimations can still be improved. Moreover, vertical deformation determination from satellite observations, such as GPS, the Gravity Recovery and Climate Experiment (GRACE) and other types of measurements, remains challenging.

In addition, seasonal mass cycles are evident around the high mountains of Tibet. In the past few years, strong seasonal fluctuations were observed by GPS and GRACE in the southern Tibetan Plateau and Nepal [9,10,11], with peak-to-peak hydrological mass variations on or near the Earth’s surface. In this study, we focus on the crustal vertical deformation in northeastern Tibet and combine GPS and GRACE observations. Comparing the annual and semi-annual amplitudes of GPS-observed and GRACE-derived seasonal displacements reveals consistent correlations. The strong seasonal consistency between GPS and GRACE will help to revise the GPS processing strategies and determine the causes of the increasing mass in northeastern Tibet.

## 2. GPS and GRACE Observations

**Figure 1:**
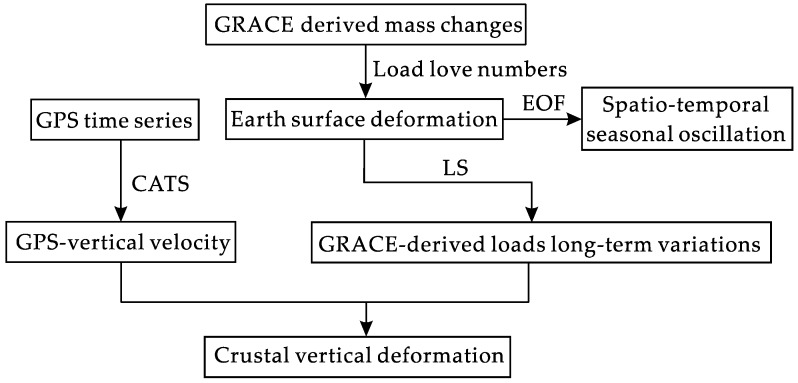
Five steps represent the brief procedures from GRACE, GPS to final crustal vertical deformation. EOF, Empirical Orthogonal Function; CATS, Create and Analyse Time Series software; LS, least squares.

GPS observes the 3D velocity in northeast Tibet. Nevertheless, crustal deformation should be corrected by surface loading effects, especially the vertical deformation. As a remote sensing technique, GRACE satellite gravimetry can observe the large-scale earth surface mass changes reported as equivalent water height [12,13]. Here, GRACE-derived surface mass loads will be used for GPS loading effects correction. Data processing procedures of GPS and GRACE are as shown in Figure 1, and detailed procedures can be found in the following sections.

### 2.1. GPS Dataset and Data Processing

**Figure 2:**
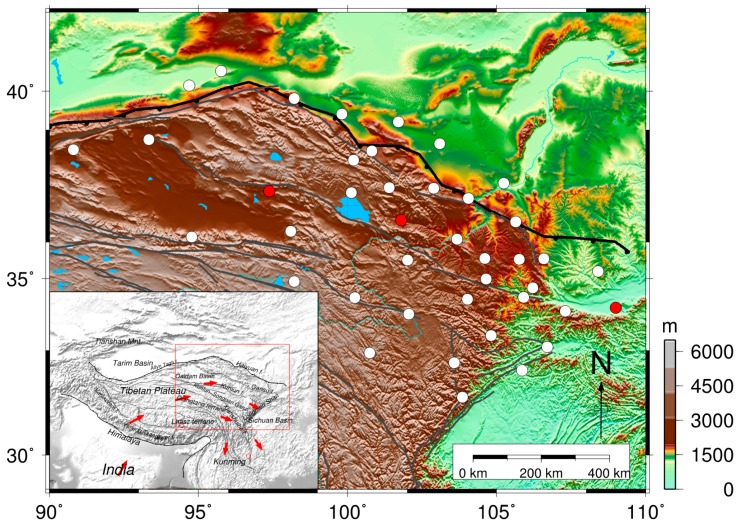
Locations of the CGPS stations in northeastern Tibet. The white circles are the CGPS stations with records spanning from March 2010 to July 2015, and the red circles denote the three stations with records spanning from January 1999 to July 2015. The map in the inset shows the geological tectonic background and crustal motions with the collision and post-collisional intra-continental deformation of the Indian and Eurasian plates. The red arrows indicate direction of large-scale block motions, and faults and subduction belts are indicated by the gray and black lines, respectively.

We used data from 41 continuous GPS (CGPS) stations (Figure 2) in the Crustal Movement Observation Network of China (CMONOC) to determine the crustal vertical deformations. Among the 41 stations, 38 have daily records for 2010–present, and three have records for 1999–present. For all CGPS stations, the record lengths exceed three years. Table 1 lists the names, geodetic latitudes and longitudes of these stations. The three-dimensional (3D) velocities derived from GPS are also given in Table 1 and will be discussed below.

We processed data from the 41 CGPSs using GAMIT/GLOBK software (version 10.40, Manufacturer, Cambridge, MA, US) [14,15] following the GPS-processing procedure described by Dong et al. [16]. The network of stations involved in this study was analyzed by tying the Earth orientation parameter (EOP) to the International Global Navigation Satellite System (GNSS) Service (IGS) orbit via phase ambiguity resolution. The Global Mapping Function (GMF) was chosen for the tropospheric mapping function and tropospheric gradients, and we used a prior dry tropospheric delay value from the Global Pressure and Temperature (GPT) model. The finite element solutions 2004 (FES2004) model with elastic Green’s Functions [17] in the reference frame of the center of mass (CM) of the whole Earth system was used to correct for ocean tides and ocean tide loading. We applied International Earth Rotation and Reference Systems (IERS) 2010 conventions to correct the tidal solid Earth and pole tides.

Loose constraints were assigned to the coordinates of each station included in the processing and to the model terms. Once the individual network solutions were obtained, the daily solutions were combined with the GLOBK software [15]. The loosely constrained solution of the complete network was then aligned by a weighted six-parameter transformation (three translation and three rotation parameters) into the 2008 International Terrestrial Reference System (ITRF2008) reference frame [18] using the coordinates and velocities of six nearby IGS stations (URUM, SHAO, LHAZ, HYDE, BJFS and KUMN), the IGS service website, and the Scripps Orbital and Position Analysis Center (SOPAC). The daily position time series of each station in the ITRF2008 reference frame was subsequently analyzed to estimate the three components of the velocity vector, the associated uncertainties and the characteristics of the time series.

The time series of all GPS sites were preprocessed to estimate the vertical velocity field. Regional GPS network common mode errors (CMEs) were removed from the GPS time series analysis using the Quali-Observation Combination Analysis (QOCA) principal component analysis (PCA) program [19,20,21]. The final slope rates were estimated by a maximum likelihood estimation (MLE) using CATS software (version, Manufacturer, Liverpool, UK) to maximize the likelihood of the observed data, including the annual and semi-annual rates, and other noises [22,23]. We use the MLE to estimate the flicker and white noise in each time series and assess their vertical velocities and uncertainties, and the final corrected rates are achieved by the GPS-derived vertical velocities subtracting the GRACE-derived uplift rates. The results are listed in Table 1.

### 2.2. GRACE Data for Mass Changes around Tibet

Mass changes deform the Earth’s surface because the Earth is an elastic body [24]. Hydrology, the atmosphere and non-tidal ocean loads contribute to this deformation, especially the vertical elastic deformation. Here, we chose the new GRACE products Level-2 Release 05 (RL5) from the Center for Space Research (CSR) of the University of Texas for the mass change and gravity computations [25]. Spherical harmonic coefficients up to degree and order 60 for the gravity field are provided monthly from April 2002 to November 2014. Because lower degree coefficients have large uncertainty, we use the result of C20 (the gravity harmonic coefficients in the degree 2 order 0), which is well constrained by the new Satellite Laser Ranging (SLR) observations [26] and the degree-1 coefficients given by Swenson et al. [27].

According to previous research works, the surface equivalent water height (EWH) can be expressed in terms of the Stokes coefficients as [28,29]:

(1) $$\Delta \sigma (\phi ,\theta )=\frac{a\rho_{e}}{3\rho_{w}}\sum_{n=0}^{\infty }\left(\frac{2n+1}{1+k_{n}}\right)\sum_{m=0}^{n}\left\{\left[\Delta C_{n}^{m}\cos(m\phi )+\Delta S_{n}^{m}\sin(m\phi )\right]{\bar{P}}_{n}^{m}(\cos\theta )\right\}$$

where *ρ_e_* and *ρ_w_* are the average density of the whole Earth and the density of water (1 g/cm^3^), respectively; *a* is the equatorial radius; *K_n_* is load Love number of degree *n*; ϕ is the latitude; *θ* is the colatitude; and $\Delta C_{n}^{m}$ and $\Delta S_{n}^{m}$ are monthly Stokes coefficients. ${\bar{P}}_{n}^{m}(\cos\theta )$ is the fully normalized Legendre function of degree *n* and order *m*.

The results of EWH secular variations in and around the Tibetan Plateau and the seasonal changes in northeastern Tibet are given in Section 3.1 based on the GRACE-derived time series processing.

## 3. Data Analysis Methods

### 3.1. GRACE-Derived Mass Change Time Series Processing

A 350-km Gaussian smoothing and P4M6 (degree 4 polynomial for order 6 of SH coefficients) de-correlation were performed to de-stripe the noise [30]. We employed a global forward modeling for removing the leakage bias in GRACE-estimated mass changes, and restoring the true magnitudes of the signal, at least on regional average basis due to truncation and spatial filtering [30,31]. GRACE products may be regarded as error free when considering the secular mass change. Further improvement of the floating mass changes could be obtained if the effect of S2 aliasing errors were excluded from the computations from GRACE data. Considering the annual and semi-annual terms and the S2 (tidal aliasing effect) 161-day period, we used least square (LS) analysis to estimate GRACE seasonal signals and trends. The LS model is as follows:

(2) $$\Delta \tilde{g}=A+Bt+\sum_{i=1}^{3}C_{i}\cos(\omega_{i}t+\varphi_{i})$$

where *A* and *B* are the constant and linear trend terms; and *C_i_*, *ω_i_* and $\varphi_{i}$ (*i* = 1, 2, 3) are the amplitude, frequency and phase of annual, semi-annual and S2 signals, respectively.

**Figure 3:**
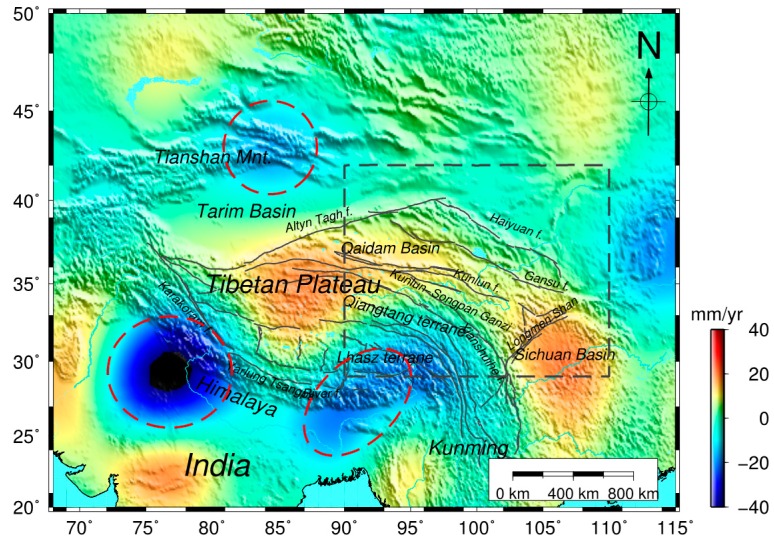
The CSR RL05 GRACE-derived mass change (equivalent water thickness) in Tibet, time spanning from April 2002 to November 2014. The mass change (kg/year) in unit square volume is expressed as equivalent water thickness (mm/year). The inset gray rectangle is our study area, mainly focusing on northeast Tibet. The India, Eastern Himalayas and Tianshan mass changes are shown in the area circled in red.

The mass changes, including soil, snow/ice and lakes are the main contributions to the GRACE-derived hydrology variations. The background shows the mass change of the EWH secular trend obtained from CSR RL05 in and around Tibet, as shown in Figure 3. The GRACE-derived mass change consists mainly of four strong signals in Tibet. The two secular variations south of Tibet show mass loss because of water resource depletion and ice melt in northern India and the Eastern Himalayas, as suggested by previous studies [10,32,33,34]. Additionally, the mass loss in Tianshan indicates that the area experiences ice melting [11]. However, few studies have focused on the mass change in northeastern Tibet using GRACE observations. Our results, as shown in Figure 3, suggest strong secular mass increase in the northeast region of Tibet.

**Figure 4:**
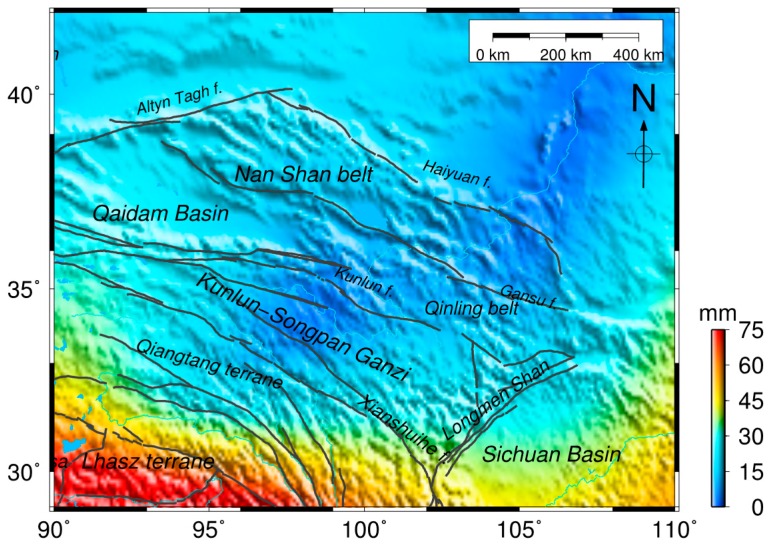
Amplitudes of seasonal signals showing peak-to-peak surface mass variations (water-equivalent height) derived from GRACE in northeastern Tibet, which is shown as the box in Figure 3. The gray solid lines indicate active major strike-slip faults.

We focus on the mass changes and crustal deformation in northeastern Tibet and divide the northeast region into elements for analysis. The amplitudes of seasonal oscillations observed by GRACE show peak-to-peak mass variations, and the corresponding signal magnitudes are between 3 and 35 mm/year in northeastern Tibet (Figure 4). These hydrological mass changes give rise to the seasonal oscillations of the Earth’s surface.

### 3.2. Empirical Orthogonal Function (EOF) Analysis of GPS Common Mode Seasonal Signals

The seasonal coordinate oscillations derived from GPS observations are largely due to surface loadings of hydrological, atmospheric and non-tidal oceanic origins [24,35,36,37]. When estimating the vertical crustal deformations by GPS, the coherence between the seasonal oscillations derived from GPS and GRACE must be evaluated. Here, this evaluation was based on the EOF, which detects the common mode seasonal oscillations in three coordinate components at the CGPS stations. As a PCA (Principal Component Analysis), the EOF analysis decomposes the coherent spatio-temporal variability of a time-variable field into a linear combination of orthogonal “modes” of standing oscillation [21,38]. The detailed theory and procedure of the EOF algorithm are described in Dong et al. [21]. Here, we briefly summarize the main procedures as follows:

1. Construct a data matrix, *D*, containing all of the GPS time series data in the selected region and time span targeted for seasonal signals.
2. Compute the covariance matrix, *R*, based on $R=\frac{1}{N-1}D\cdot D^{T}$, where *N* is the number of observations.
3. Diagonalize the eigenvalues and eigenvectors of *R*, *RK* = *KΛ*, where *Λ* is a diagonal matrix containing the eigenvalues *λ_i_* of *R*, and *K* consists of the eigenvectors *k_i_* of *R* in the form of column vectors.
4. Decompose the data matrix into different orthogonal “modes of variability” by arranging the eigenvectors in descending order of eigenvalues.
5. Obtain the principal components (PCs).

The PCs represent the dominant time-variations, and the eigenvectors represent the corresponding spatial responses to the PCs of all CGPS sites. The variance is defined by the eigenvalue of each mode. The eigenvalues indicate the amounts of variance explained by each eigenvector. The sum of all of the eigenvalues equals the total variance in the original data. The first EOF is defined as that accounting for the maximum amount of variance in the dataset [39].

**Figure 5:**
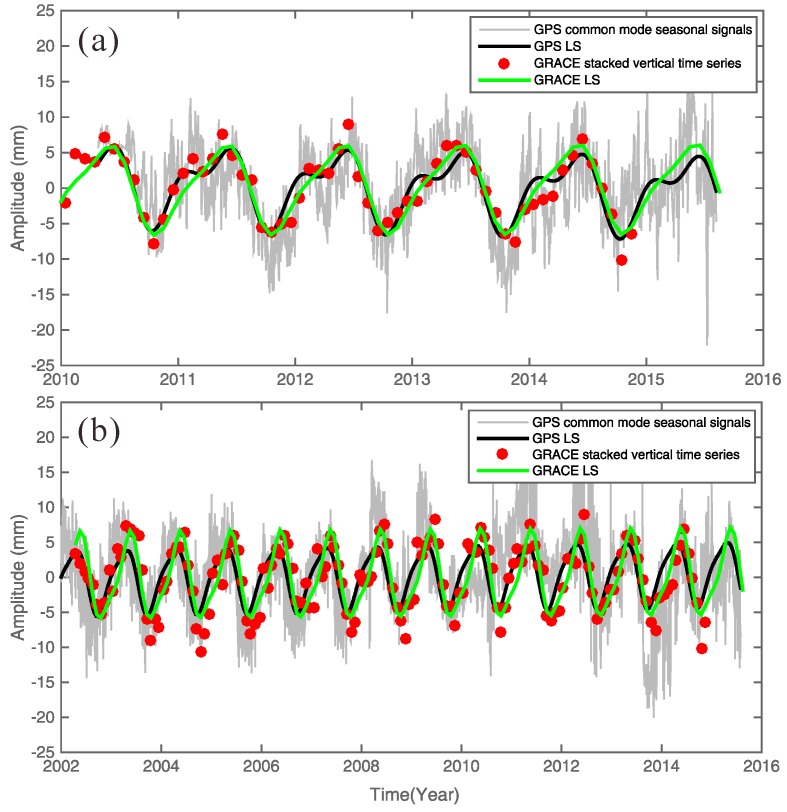
The first common mode from the EOF corresponding to the seasonal signals based on (**a**) the records from 2010 to 2015 of 38 GPS stations, and (**b**) the three CGPS sites records are from 1999 to 2015. In order to compare with GRACE data (data records from 2002 to 2015), we just selected GPS data from 2002 to 2015. GRACE monthly measurements span from April 2002 to November 2014. Gray lines (daily solutions) and red dots (monthly solutions) correspond to the GPS and GRACE results, which are LS fitted by the expression in Equation (2) (i.e., the black and green lines, respectively).

Higher-order PCs are usually related to local or individual site effects. That is, the first few PCs represent the dominant contributors to the variability in all time series in the network and usually arise from the same origins. Figure 5 shows the common seasonal signals from the EOF at all of the GPS sites. The EOF results are described and analyzed in Section 4.1. The first few PCs represent the largest contributors to the variance in the network residual time series and are usually related to the common source time function.

## 4. Results and Discussion

### 4.1. Seasonal Oscillations around Northeastern Tibet

Continental water-storage changes alter the surface loading and lead to surface deformations dominated by annual oscillations [40]. GPS may detect strong seasonal surface deformations in northeastern Tibet (Figure 5). To estimate the seasonal surface deformation due to the mass load effect, we use the CSR-provided De-aliasing Level-1B (AOD1B) solution (GAC products) and the monthly Stokes coefficient GSM (GRACE Satellite only Model) to compute the whole surface vertical loads, including the hydrological, atmospheric and non-tidal ocean loads. The products were applied to the elastic displacement caused by the changing surface mass loads [41]:

(3) $$\Delta h(\theta ,\phi )=R\sum_{l=1}^{\infty }\sum_{m=0}^{l}{\bar{P}}_{lm}(\cos\theta )\cdot [C_{lm}\cos(m\phi )+S_{lm}\sin(m\phi )]\cdot \frac{h_{l}^{'}}{1+k_{l}^{'}}$$

where R is the Earth’s radius; ${\bar{P}}_{lm}$ is the fully normalized Legendre function for degree l and order m; $C_{lm}$ and $S_{lm}$ are the spherical harmonic coefficients of the gravity field; $h_{l}^{'}$ and $k_{l}^{'}$ are adopted load Love numbers provided by Farrell [17] and computed relative to the CM of solid Earth [9]. In Figure 5, the red dots are the GRACE-derived mass loads (including the hydrological, atmospheric and non-tidal ocean loads) causing surface deformation, which constitute de-trended stacked average loading deformation time series corresponding to GPS site locations.

The GPS and GRACE data series clearly display very similar and consistent seasonal patterns in terms of both the magnitude and the phase. We compute the GPS seasonal variations by LS fitting with annual and semiannual periodic terms and then compare them with the seasonal variations observed by GRACE, as shown in Figure 5. The GPS common mode time series in northeastern Tibet shows significant seasonal variations in which the peak-to-peak amplitude can exceed one centimeter.

Here, we compare the consistency between GPS common mode seasonal signals and GRACE stacked-average seasonal signals. First, we removed the GRACE-derived seasonal vertical deformations from the GPS-observed, de-trended height time series and computed the reductions in the weighted root-mean-squares (WRMS) as follows [9,36,42]:

(4) $$WRMS_{reduction}=\frac{WRMS_{GPS\_LS}-WRMS_{GPS\_LS-GRACE\_LS}}{WRMS_{GPS\_LS}}$$

(5) $$WRMS=\sqrt{\frac{\frac{n}{n-1}\sum_{i=1}^{n}\frac{{\left(P_{i}-P\right)}^{2}}{\sigma_{i}^{2}}}{\sum_{i=1}^{n}\frac{1}{\sigma_{i}^{2}}}}$$

where n is the number of days, $P_{i}$ is the estimate of the component on the *i-*th day, P is the weighted average of the component estimate over all days, and $\sigma_{i}$ is the formal error. $WRMS_{reduction}$ = 1.0 indicates perfect agreement between GPS-observed and GRACE-modeled annual plus semiannual displacements.

The WRMS Reduction Ratio of common mode seasonal signals was 0.90 for 38 GPS stations and 0.81 for three long-term GPS stations. The consistency between the GPS common mode signals and GRACE stacked average seasonal signals demonstrates that the seasonal position oscillations in northeastern Tibet are mainly caused by mass loading changes, including the hydrological, atmospheric and non-tidal ocean loads.

### 4.2. Vertical Crustal Deformation

As an elastic body, the Earth’s surface moves upward in response to a loss in loading and downward as the loading increases. In addition to the evident seasonal oscillations described in Section 4.1, a general subsiding trend was also predicted in northeastern Tibet by the least squares method. It is assumed that Glacial Isostatic Adjustment (GIA) is absent and that all tectonic motions maintain isostatic equilibrium. The secular change in the gravity field is due to the present-day surface mass changes [10], without considering GIA or tectonic processes, such as crustal thickening.

**Figure 6:**
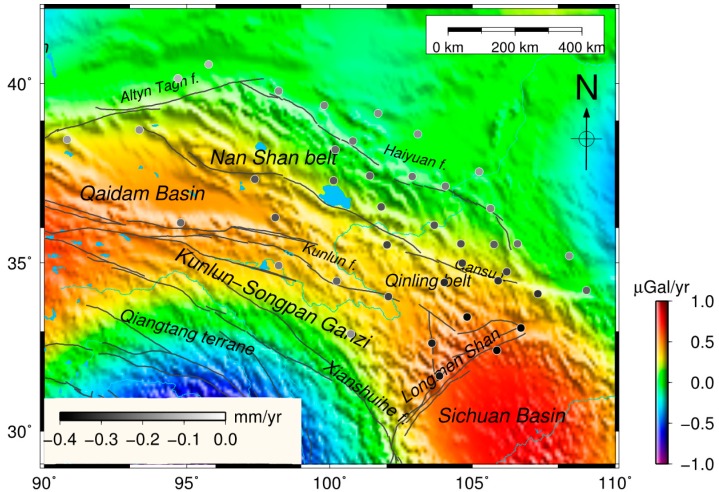
GRACE mass change in Tibet. The CSR RL5 was used to estimate the gravity in northeastern Tibet, spanning from April 2002 to November 2014. The dots denote the vertical deformations caused by surface loads corresponding to the GPS sites. The color bars internal and external denote the rates of the GRACE derived loading and gravity variations, respectively.

As shown in Figure 6, we compute the GRACE-derived long-term loadings using the trend from the CSR solutions corresponding to all CGPS sites used in this paper. The least squares method is used to estimate the trend rates of all of the sites, while considering the annual and semi-annual signals in calculation. The surface deformation shows a subsidence rate of approximately −0.1 to −0.4 mm/year because of the increased loadings in northeast Tibet. As the mass increases southeastward, the inner rates of the load slopes gradually decrease with increasing mass. However, outside of this region, deformation exerts a weaker effect on the load. The maximum load-induced vertical deformation is approximately −0.4 mm/year in the Longmen Shan fault zone. This is caused by the compression between the Sichuan Basin and the northwest region of Tibet that is increasing in mass.

**Figure 7:**
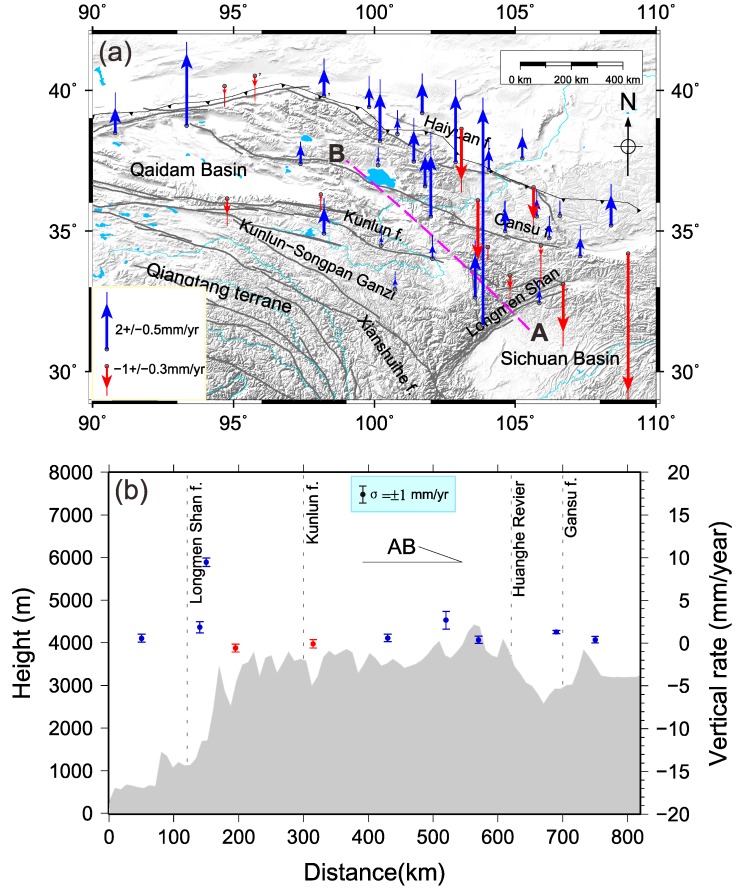
(**a**) the vertical crustal deformation rates in northeastern Tibet without the loading effect (from GRACE). Blue vectors denote uplift, and red vectors denote subsidence. The active faults in Tibet are marked by gray lines, and the black lines with triangles show two subduction belts in the northern and southern boundaries of Tibet; (**b**) profile charts showing the terrain height and uplift rate of the northeast Tibetan Plateau, the magenta dotted line from A to B in (**a**). The blue dots with error bars denote GPS sites with positive uplift rates and the red dots denote negative rates. The error bars represent one standard deviation on either side of the plotted points.

After removing the GRACE-derived loading effects, we obtained the vertical crustal velocities from GPS that represent the vertical crustal motions of the northeast region of the Tibetan Plateau, as shown in Figure 7a. The corrected vertical crustal deformations show that northeastern Tibet undergoes uplift because of the compression of the bilateral crustal blocks. The active subduction of the north China craton is responsible for crustal depression [43]. The crustal uplift and subsidence are anisotropic in northeastern Tibet. Some regional sites show both uplift and subsidence because of internal tectonic deformation. For example, the northern margin of the Qaidam Basin, which is at the edge of the subduction zone, is uplifted due to the oblique push from the Tarim massif to Tibet. In contrast, the southern boundary undergoes crustal subsidence attributed to the asthenosphere in the basin boundary.

Active faults are often accompanied by complex tectonic movements. The vertical deformation rate in the Longmen Shan active fault zone is 9.468 mm/year, which is the maximum in northeastern Tibet. In Figure 7b, we can quantitatively see the unusual uplift of the Longmen Shan fault zone. From the crustal structure image, Yangtze (e.g., the Sichuan basin) sub-continent crust extends beneath the eastern Tibetan Plateau and it implies the uplift of the Longmen Shan range [44]. The AB profile shows that the terrain height increases dramatically from 1200 m to 4000 m. The Kunlun strike–slip fault shows the negative uplift rates, which indicate that the block is sinking with the movement of internal material of the northeast Tibetan Plateau [45].

### 4.3. Contribution of Non-Tectonic Processes to Vertical Crustal Deformation

GRACE observations of mass change are limited by poor spatial resolution. The regional mass change will contribute to the earth surface deformation due to mass loading effects. The loading effects (including the atmosphere, non-tidal and hydrology) in northeast Tibet are about −0.1 mm/year to −0.4 mm/year due to the continuous mass increase. Here, we use the interpolation method to obtain the variation of loads deformation in northeast Tibet corresponding to the GPS locations by using GRACE observation. However, the consistency of spatio-temporal GPS and GRACE time series in northeast Tibet indicating the seasonal oscillation is mainly caused by earth surface loading effects.

Scientists have paid close attention to whether the observed crustal uplift may potentially affect the GRACE gravity measurement. Previous studies have reported that the tectonic uplift of the Tibetan Plateau is too slow to severely interrupt the isostatic equilibrium process, and thus, it may exert only a small effect on the gravity change [9,10,46]. GRACE observations indicate that the gravity changes in the northeast region of the Tibetan Plateau are mainly attributable to increasing surface hydrological mass and that those changes can be safely interpreted in terms of surface mass loads [47,48]. However, a likely scenario might be that any broad-scale tectonic uplift would be isostatically compensated by an increasing mass deficiency at depth, with little net effect on gravity and consequently without noticeable contribution to the GRACE results [48]. Therefore, we can ignore the uplifting tectonic impacts on gravity changes in the GRACE measurements.

Royden et al. [49] reported that eastern Tibet has undergone rapid surface uplift and crustal thickening. In this study, Figure 6 and Figure 7a suggest that the mass is increasing gradually, whereas the crust is uplifting in northeastern Tibet. Nevertheless, we obtained the vertical crustal deformation without considering possible effects of GIA, which could contribute to both gravity changes and vertical motion [9]. Whether the loss of the ice sheet of Tibet will lead to significant crustal uplift in this area remains controversial [10,50]. The GIA effect in Xiang et al. [34] was computed based on the ICE-6G_C (VM5a) model [51,52] which was constrained by space geodesy, such as GPS data. Though it is reliable and useful in America and Antarctica, there were no sufficient GPS data in Tibet when they established the ice-age terminal deglaciation. Additionally, the long-term gravity change related to tectonic isostatic equilibrium should be of little consequence to the cryospheric effect. Therefore, we ignored the effect of GIA on gravity changes when we used GRACE data to obtain the mass change. The corrected vertical velocity determined using the GPS and GRACE measurements was solely caused by tectonic movement.

The rapid changes in polar motion since 2005 have resulted in large-scale elastic radial deformations of the Earth in some local regions. Vertical velocities derived from geodetic techniques are affected by rapid changes in polar motions [53,54], which affect the periodic terms rather than the trends of the deformation derived from GPS.

## 5. Conclusions

Mass changes in Tibet are highly complex. GRACE observations indicate strong seasonal hydrological oscillations of the Earth surface in and around the Tibetan region. The seasonal mass changes will contribute to surface load deformation resulting from redistributions of the fluid mass loads, as observed by CGPS measurements in northeastern Tibet. Comparing the EOF-decomposed common mode seasonal signals and GRACE-derived load stacked average series revealed strong consistency, indicating that the surface mass loads are the main contributors to the surface deformation.

In addition to the dominating seasonal signals, trends in GRACE-derived mass changes are evident and can be attributed to gradual accumulations of crustal materials caused by plate collisions (Figure 6). The subduction of the Indian plate beneath the Eurasian plate and the pushing of the Eurasian Plate along the Himalayas in the NNE direction may have caused the Indian plate to rotate clockwise around the Eastern Himalayan Syntaxis, resulting in a fan-like front at the southeast corner of the Tibet plateau. The GRACE-derived mass changes confirm this clockwise rotation with an increasing mass anomaly towards the southeast (Figure 3). This interesting pattern in the GRACE data warrants thorough investigation, but this is beyond the scope of this study.

The uplift of northeastern Tibet, indicated by GPS after removing the GRACE-derived long-term rate, is due to secular changes in loading of tectonic origin (Figure 7). This uplift might have resulted from the compression of the surrounding crustal blocks, which also causes mass accumulation, as seen in the GRACE-derived mass change. By contrast, sites undergoing subsidence are located along the Qaidam basin boundary, which could be caused by the active subduction of the North China craton. The northeast Tibetan Plateau is still in a growing process due to the Asian lithosphere underthrusting beneath the northeastern Tibetan Plateau.

## Figures and Tables

**Table 1:** GPS stations with their vertical velocities, and GRACE-derived uplift rates.

Site	Latitude (°)	Longitude (°)	Durations	GPS-Derived Velocity (mm/year)	GRACE-Derived Uplift (mm/year)	Corrected Vertical Rate (mm/year)
DLHA	37.38	97.37	1999–2015	0.55 ± 0.15	−0.26 ± 0.04	0.81 ± 0.16
GSAX	40.51	95.76	2010–2015	−0.61 ± 0.60	−0.12 ± 0.04	−0.49 ± 0.60
GSDH	40.14	94.68	2010–2015	−0.48 ± 0.54	−0.11 ± 0.04	−0.37 ± 0.54
GSJY	39.80	98.21	2010–2015	0.99 ± 0.46	−0.18 ± 0.04	1.18 ± 0.46
GSGL	37.45	102.88	2010–2015	2.73 ± 0.65	−0.22 ± 0.04	2.95 ± 0.65
GSGT	39.41	99.81	2010–2015	0.67 ± 0.49	−0.19 ± 0.04	0.86 ± 0.49
GSML	38.44	100.81	2010–2015	0.42 ± 0.41	−0.22 ± 0.04	0.63 ± 0.41
GSMQ	38.63	103.08	2010–2015	−2.42 ± 0.54	−0.18 ± 0.04	−2.24 ± 0.54
GSGL	37.45	102.88	2010–2015	2.73 ± 0.65	−0.22 ± 0.04	2.95 ± 0.65
GSJT	37.18	104.05	2010–2015	0.67 ± 0.49	−0.21 ± 0.04	0.87 ± 0.49
GSDX	35.55	104.60	2010–2015	0.03 ± 0.30	−0.27 ± 0.05	0.31 ± 0.30
GSJN	35.52	105.75	2010–2015	0.50 ± 0.41	−0.25 ± 0.05	0.75 ± 0.41
GSPL	35.54	106.58	2010–2015	0.00 ± 0.44	−0.23 ± 0.05	0.22 ± 0.44
GSLX	34.99	104.64	2010–2015	0.64 ± 0.40	−0.30 ± 0.051	0.94 ± 0.40
GSMX	34.43	104.02	2010–2015	−0.43 ± 0.47	−0.31 ± 0.05	−0.10 ± 0.47
GSMA	34.01	102.05	2010–2015	0.30 ± 0.42	−0.29 ± 0.04	0.60 ± 0.42
GSWD	33.42	104.81	2010–2015	−0.95 ± 0.44	−0.37 ± 0.05	−0.58 ± 0.44
GSTS	34.48	105.91	2010–2015	−0.74 ± 1.19	−0.32 ± 0.05	−0.42 ± 1.19
GSQS	34.74	106.21	2010–2015	0.31 ± 0.37	−0.29 ± 0.05	0.60 ± 0.37
NMAY	39.20	101.70	2011–2015	1.13 ± 0.40	−0.18 ± 0.04	1.32 ± 0.40
NXZW	37.58	105.24	2010–2015	0.82 ± 0.30	−0.16 ± 0.04	0.98 ± 0.30
NXHY	36.55	105.64	2010–2015	−1.60 ± 0.29	−0.19 ± 0.05	−1.41 ± 0.29
QHLH	38.74	93.33	2010–2015	2.99 ± 0.50	−0.17 ± 0.04	3.16 ± 0.50
QHMY	38.47	90.80	2011–2015	0.94 ± 0.67	−0.16 ± 0.04	1.10 ± 0.67
QHQL	38.20	100.20	2011–2015	1.85 ± 0.61	−0.23 ± 0.04	2.08 ± 0.61
QHGE	36.14	94.77	2010–2015	−0.91 ± 0.50	−0.24 ± 0.04	−0.68 ± 0.50
QHDL	36.29	98.09	2010–2015	−0.53 ± 0.49	−0.27 ± 0.04	−0.26 ± 0.49
QHME	37.47	101.40	2010–2015	1.09 ± 0.56	−0.24 ± 0.04	1.34 ± 0.56
QHGC	37.33	100.13	2010–2015	0.137 ± 0.37	−0.26 ± 0.04	0.40 ± 0.37
QHMD	34.92	98.20	2010–2015	0.91 ± 0.41	−0.21 ± 0.04	1.12 ± 0.41
QHMQ	34.47	100.24	2010–2015	0.13 ± 0.42	−0.25 ± 0.04	0.37 ± 0.42
QHTR	35.51	102.01	2010–2015	2.38 ± 1.03	−0.29 ± 0.04	2.67 ± 1.03
QHBM	32.93	100.74	2010–2015	0.29 ± 0.33	−0.17 ± 0.04	0.46 ± 0.33
SCMX	31.67	103.85	2010–2015	9.11 ± 0.49	−0.36 ± 0.05	9.47 ± 0.49
SCSP	32.64	103.58	2010–2015	1.50 ± 0.64	−0.35 ± 0.05	1.84 ± 0.64
SCGY	32.43	105.85	2010–2015	0.11 ± 0.47	−0.44 ± 0.05	0.55 ± 0.47
SNMX	33.10	106.70	2011–2015	−2.49 ± 0.61	−0.41 ± 0.05	−2.08 ± 0.61
SNXY	35.20	108.40	2010–2015	1.15 ± 0.48	−0.18 ± 0.05	1.33 ± 0.48
SNTB	34.10	107.30	2011–2015	0.49 ± 0.54	−0.32 ± 0.05	0.81 ± 0.54
XIAA	34.20	109.00	1999–2015	−6.29 ± 0.41	−0.24 ± 0.05	−6.05 ± 0.41
XNIN	36.60	101.80	1999–2015	1.03 ± 0.17	−0.27 ± 0.04	1.30 ± 0.17
